# Oncostatin M promotes osteogenic differentiation of tendon-derived stem cells through the JAK2/STAT3 signalling pathway

**DOI:** 10.1186/s13018-024-04915-5

**Published:** 2024-07-16

**Authors:** Jun Yang, Xiaolin Chen, Yueshu Wu, Gang Xu, Xiaochen Qu

**Affiliations:** 1 Department of Orthopaedics, First Affiliated Hospital of Dalian Medical University, Dalian, PR China; 2 Department of Orthopedic Surgery, The Second Affiliated Hospital of Chongqing Medical University, No. 76, Linjiang Road, Chongqing, 400010 Yuzhong District PR China

**Keywords:** Osteogenic differentiation, JAK2/STAT3 signalling, Oncostatin M, Tendon-derived stem cells

## Abstract

**Purpose:**

Oncostatin M (OSM) is involved in the regulation of osteogenic differentiation and has a major role in the development of heterotopic ossification. The role of OSM in osteogenic differentiation of tendon-derived stem cells (TDSCs) and its mechanism have not been reported. This study aim to investigate the role of OSM in osteogenic differentiation of TDSCs and study the mechanism.

**Methods:**

TDSCs were differentiated in osteogenic differentiation medium for 7 days. Recombinant OSM was added to the osteogenic differentiation medium for 7 and 14 days. The effect of Janus kinase 2 (JAK2) inhibitor AZD1480 and signal transducer and activator of transcription 3 (STAT3) inhibitor stattic in the presence of recombinant OSM on osteogenic differentiation of TDSCs was examined after differentiation for 7 and 14 days. Alkaline phosphatase and alizarin red staining were used to assess the effects on early and mid-stage osteogenic differentiation, respectively. Western blotting and qPCR were used to assess the expression of receptor and signalling pathway-related proteins and osteogenic marker genes, respectively.

**Results:**

TDSCs were successfully induced to differentiate into osteoblasts. Recombinant OSM promoted osteogenic differentiation of TDSCs to early and mid-stages. After addition of AZD1480 or stattic, decreased alkaline phosphatase and alizarin red staining were observed in the early and mid-stages of osteogenic differentiation. Additionally, decreased expression of receptor and pathway-related proteins, and osteogenic genes was found by western blotting and qPCR, respectively.

**Conclusion:**

OSM promotes osteogenic differentiation of TDSCs and the JAK2/STAT3 signalling pathway plays an important role.

## Introduction

Tendon heterotopic ossification is a pathological bone formation that occurs at a tendon site, typically at a joint, with a higher probability of heterotopic ossification at the elbow joint after injury [1]. Tendon heterotopic ossification is often accompanied by local pain, joint movement limitation, and other symptoms, which have a serious adverse effect on quality of life [2]. Surgery is the main treatment for tendon heterotopic ossification, but additional trauma occurs easily during surgery, which leads to recurrence of tendon heterotopic ossification [3]. Therefore, the pathogenesis of tendon heterotopic ossification needs to be explored to improve its treatment.

After an injury to tendon tissue occurs, the cells within the tendon tissue can differentiate normally into tendon cells for repair [4, 5]. However, most of the injury sites develop tendon heterotopic ossification.Tendon heterotopic ossification is thought to be related to dormant osteogenic precursor cells in tendon tissue. Upon tendon tissue stimulation, its osteogenic precursor cells differentiate into osteoblasts and form bone-like material, which in turn develops into heterotopic ossification [6]. The biological functions of tendon stem cells are similar to those of mesenchymal stem cells (MSCs) with self-renewal and a multidirectional differentiation potential for cartilage cells, osteoblasts, and adipocytes, which participate in tissue regeneration and repair of tendon injuries [7–9]. Osteogenic differentiation of tendon stem cells plays a major role in tendon heterotopic ossification [10, 11].

The role of the JAK/STAT signalling pathway in osteogenic differentiation has been extensively studied. The inflammatory environment also has an important role in osteogenic differentiation, and macrophages are critical regulators of inflammation. Under non-coculture conditions, macrophage-associated inflammatory factors TNF-α, IL-6, IL-1β, and OSM are involved in the regulation of osteogenic differentiation with OSM promoting osteogenic differentiation more significantly [12, 13]. Although some studies have reported on OSM in osteogenic differentiation, the role of OSM and JAK/STAT signalling in osteogenic differentiation of TDSCs remains unclear.

This study aimed to elucidate the role of OSM in osteogenic differentiation of TDSCs and its mechanism. We found that OSM promoted osteogenic differentiation of TDSCs through the JAK2/STAT3 signalling pathway.

## Materials and methods

### Cell culture and osteogenic differentiation

TDSCs(Sourced from rats)were purchased from Percell (Life Science and Technology Ltd., Wuhan, China). Primary tendon stem cells were cultured in 100 mm dishes containing Dulbecco’s modified Eagle’s medium (DMEM, GIBCO) supplemented with 10% fetal bovine serum (GIBCO), 100 U/ml penicillin, and 100 mg/ml streptomycin at 37 °C with 5% CO_2_ in a humidified atmosphere. Primary cells were passaged using 0.25% trypsin. Second and third passage cells were used in experiments.

For osteogenic differentiation, cells were cultured in osteogenic medium consisting of DMEM supplemented with 50 µM ascorbic acid (Sigma-Aldrich, St. Louis, MO), 10 mM glycerophosphate (Sigma-Aldrich), and 10 nM dexamethasone (Sigma-Aldrich).

### Cell treatments

Cells were seeded in six-well plates at 1 × 10^5^ cells/well and cultured at 37 °C with 5% CO_2_. At 70% confluence, the medium was replaced with 2 mL osteogenic differentiation medium. Medium changes were performed every 2–3 days.

To determine whether TDSCs could differentiate into osteoblasts, TDSCs were stimulated for 7 days in osteogenic differentiation medium.

To investigate the effect of OSM on the JAK2/STAT3 signalling pathway and osteogenic differentiation of TDSCs, we added 25 ng/ml OSM (P6381, Abnova) to the osteogenic differentiation medium. Osteogenic differentiation medium without recombinant OSM was used as a control. Differentiation was continued for 7 and 14 days.

To further analyse the effect of JAK2/STAT3 signalling on osteogenic differentiation of TDSCs, JAK2 inhibitor AZD1480 (10 µM, Santa Cruz Biotechnology, Dallas, TX), STAT3 inhibitor stattic (5 µM, Santa Cruz Biotechnology), or DMSO was added to the osteogenic differentiation medium with 25 ng/mL recombinant OSM to differentiate TDSCs for 7 and 14 days.

### Quantitative real-time polymerase chain reaction (qRT-PCR) analysis

Expression levels of osteogenic marker genes in TDSCs were measured by qRT-PCR. Total RNA was isolated using Trizol (Invitrogen, Carlsbad, CA). Reverse transcription and qPCR were performed using a BeyoFast™ SYBR Green One-Step qRT-PCR Kit (D7268; Beyotime, Shanghai, China) in accordance with the manufacturer’s instructions on an IQ5 system (Bio-Rad, Hercules, CA). Values were normalised to glyceraldehyde-3-phosphate dehydrogenase (GAPDH) expression using the 2-^ΔΔCt^ method. Primer sequences are provided in Table 1.

**Table 1:** Primer sequences for quantitative real-time polymerase chain reaction (qRT-PCR).

Gene	Primer (5′–3′)	
	Fw	Rv
ALP	AGATGGATGAGGCCATCGGA	CCAAACGTGAAAACGTGGGA
RUNX2	CAGACCAGCAGCACTCCATA	AGACTCATCCATTCTGCCGC
Osterix	GGTCCTGGCAACACTCCTAC	AAGAGGTGGGGTGCTGGATA
OCN	CTGAGTCTGACAAAGCCTTC	GCTGTGACATCCATACTTGC
OPN	CAGTCGATGTCCCTGACGG	GTTGCTGTCCTGATCAGAGG
GAPDH	CAGGGCTGCCTTCTCTTGTG	GATGGTGATGGGTTTCCCGT

### Western blot analysis

Cell lysates were prepared using radioimmunoprecipitation buffer (Beyotime) containing 10 mM phenylmethylsulphonyl fluoride (Beyotime) as a protease inhibitor. Western blot analyses were carried out as described previously [14]. The following primary mouse anti-rat monoclonal antibodies were purchased from Santa Cruz Biotechnology: OSMR (1:500, sc-376,511) and GP130 (1:500, sc-376,280). The following primary rabbit anti-rat monoclonal antibodies were purchased from Abcam (Cambridge, UK): JAK2 (1:4000, ab108596), p-JAK2 (1:5000, ab32101), STAT3 (1:1500, ab68153), p-STAT3 (1:5000, ab32143), and β-catenin (1:5000, ab32572). Immunoreactive bands were semi-quantified using ImageJ software and normalised to corresponding β-actin bands.

### Alkaline phosphatase (ALP) activity staining

TDSCs were induced to differentiate into osteoblasts using osteogenic differentiation medium. Briefly, 5 × 10^4^ TDSCs were seeded in 12-well plates. At 70% confluence, the cells were cultured in osteogenic differentiation medium containing OSM and inhibitors for 7 days. Osteoblasts were fixed with 4% paraformaldehyde for 25 min, the fixative was discarded and washed twice with PBS, followed by staining with alkaline phosphatase chromogenic kit (Beyotime) for 20 min, the staining solution was discarded and washed twice with PBS, and the staining was then transferred to the microscope for observation.

### Alizarin red staining

Alizarin red staining was used to observe mineral deposition after osteogenic differentiation of TDSCs. TDSCs were seeded in 12-well plates at 5 × 10^4^ cells/well. At 70% confluence, the cells were stimulated with osteogenic differentiation medium containing OSM and inhibitors for 2 weeks. The cells were then fixed with 4% paraformaldehyde for 25 min and then stained with 0.1% alizarin red (Sigma-Aldrich) for 20 min.

### Statistical analysis

Statistical analyses were performed using SPSS version 13.0. Student’s t-test was used for comparisons between two groups. For comparisons between multiple groups, one-way analysis of variance (ANOVA) and the least significant difference (LSD) test were performed. Data are expressed as means ± standard error. *p* < 0.05 was considered significant.

## Results

### Osteogenic differentiation of TDSCs

**Fig. 1:**
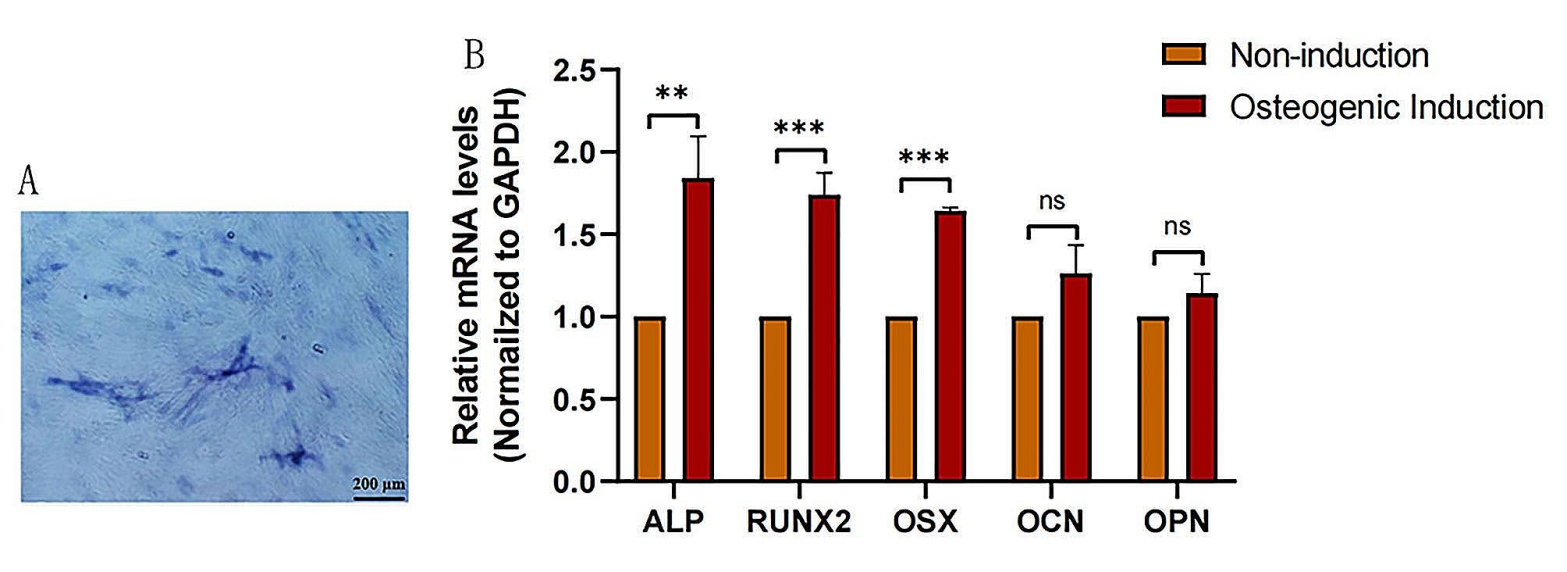
Osteogenic differentiation of tendon stem cells. (A) Alkaline phosphatase staining of tendon stem cells after osteogenic differentiation. Scale bar represents 200 μm. (B) qPCR assays of osteogenic marker gene expression at 7 days of osteogenic differentiation (*n* = 3). Two-tailed Student’s t-test. Data are presented as means ± standard deviation. *p < 0.05; **p < 0.01; ***p < 0.001

To investigate whether TDSCs could differentiate into osteoblasts, the cells were differentiated in osteogenic differentiation medium for 7 days, and then the expression of osteogenic marker genes in the differentiated TDSCs was measured by qPCR. As shown in Fig. 1A, the cells were positive for alkaline phosphatase staining after 7 days in osteogenic differentiation medium. Additionally, the expression of osteogenic marker genes ALP, RUNX2, and OSX was significantly increased in the differentiated group compared with the control group (Fig. 1B). Therefore, the tendon stem cells had differentiated into osteoblasts.

### OSM promotes early and mid-stage osteogenic differentiation of TDSCs

**Fig. 2:**
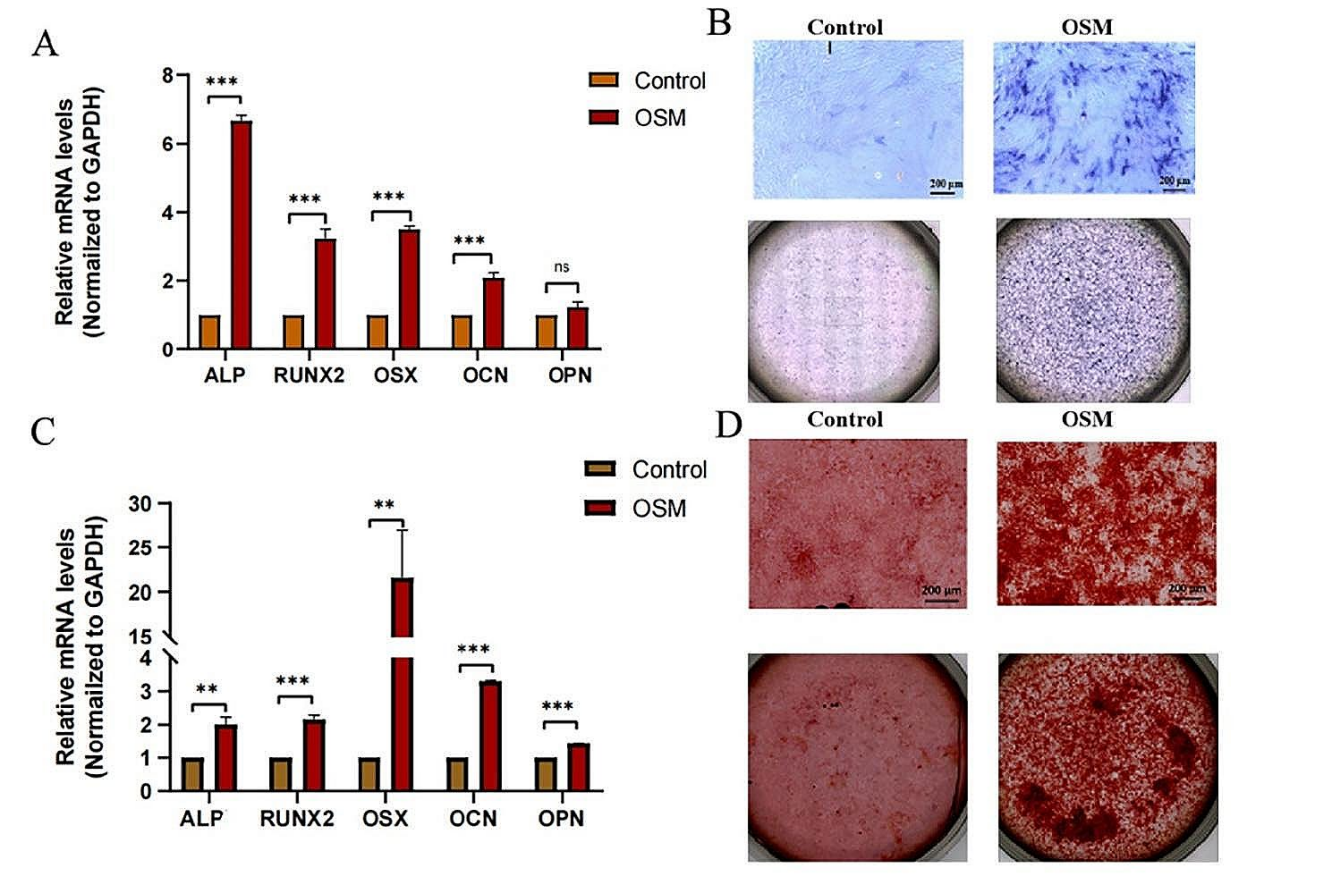
OSM promotes early and mid-stage osteogenic differentiation of tendon stem cells. Figures A and C show osteogenic differentiation of tendon stem cells in the presence of OSM for 7 and 14 days, respectively, and the expression of osteogenic marker genes detected using qPCR (*n* = 3). Two-tailed Student’s t-test. OSM induced osteogenic differentiation of tendon stem cells after 7 and 14 days, Figures B and D are alkaline phosphatase staining and alizarin red staining, respectively. Scale bar represents 200 μm. Data are presented as means ± standard deviation. *p < 0.05; **p < 0.01; ***p < 0.001

To explore the effects of OSM on early and mid-stage osteogenic differentiation of TDSCs, we added OSM to the osteogenic differentiation medium and cultured the TDSCs for 7 and 14 days. As a result, the expression of osteogenic marker genes ALP, RUNX2, OSM, OCN, and OPN was significantly upregulated in the experimental group relative to the control group (Fig. 2A and C). Alkaline phosphatase and alizarin red staining were performed at 7 and 14 days of TDSC differentiation, respectively. Alkaline phosphatase staining of the OSM group was stronger than that of the control group, and alizarin red staining indicated that OSM increased mineralisation after osteogenic differentiation of TDSCs (Fig. 2B and D). Taken together, these results showed that OSM promoted early and mid-stage osteogenic differentiation of TDSCs.

### OSM promotes early and mid-stage osteogenic differentiation of TDSCs via the JAK2/STAT3 signalling pathway

**Fig. 3:**
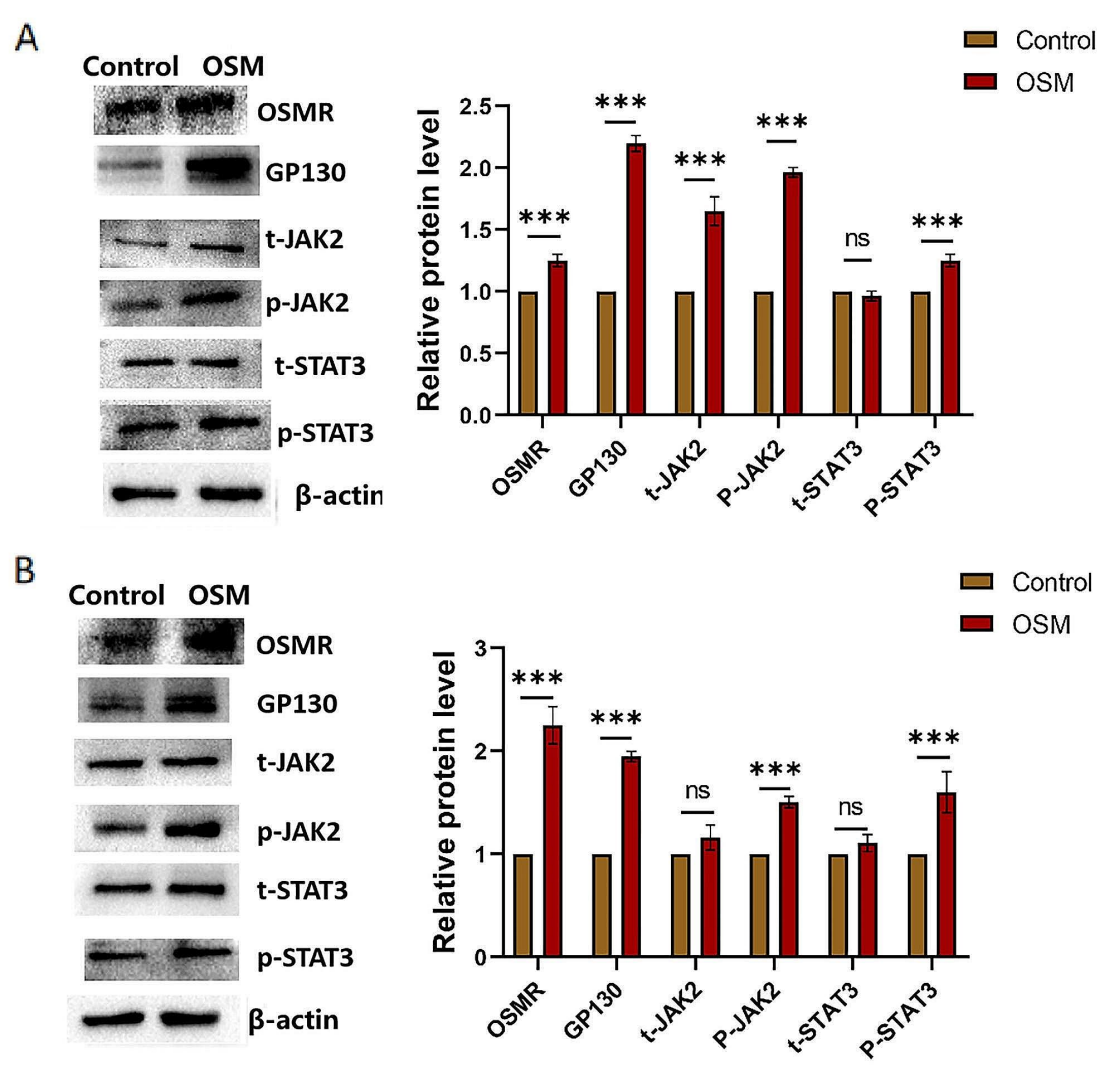
Effect of OSM on JAK2/STAT3 signalling pathway during early and middle osteogenic differentiation of tendon stem cells. (A) After OSM-induced osteogenic differentiation of tendon stem cells for 7 days, expression of OSMR, GP130, p-JAK2, and p-STAT3 proteins was detected by western blotting (*n* = 3). Two-tailed Student’s t-test. (B)Fourteen days after OSM-induced osteogenic differentiation of tendon stem cells, expression of OSMR, GP130, p-JAK2, and p-STAT3 proteins was detected by western blotting (*n* = 3). Two-tailed Student’s t-test.Data are presented as means ± standard deviation. *p < 0.05; **p < 0.01; ***p < 0.001

To analyse the effect of OSM on the JAK2/STAT3 signalling pathway during osteogenic differentiation of TDSCs, the protein expression of receptors OSMR and GP130 as well as JAK2 and STAT3 was detected by western blotting after 7 and 14 days of osteogenic differentiation of TDSCs. The results showed that expression of OSMR, GP130, p-JAK2, and p-STAT3 proteins was significantly increased in the OSM group compared with the control group (Fig. 3A and B).

**Fig. 4:**
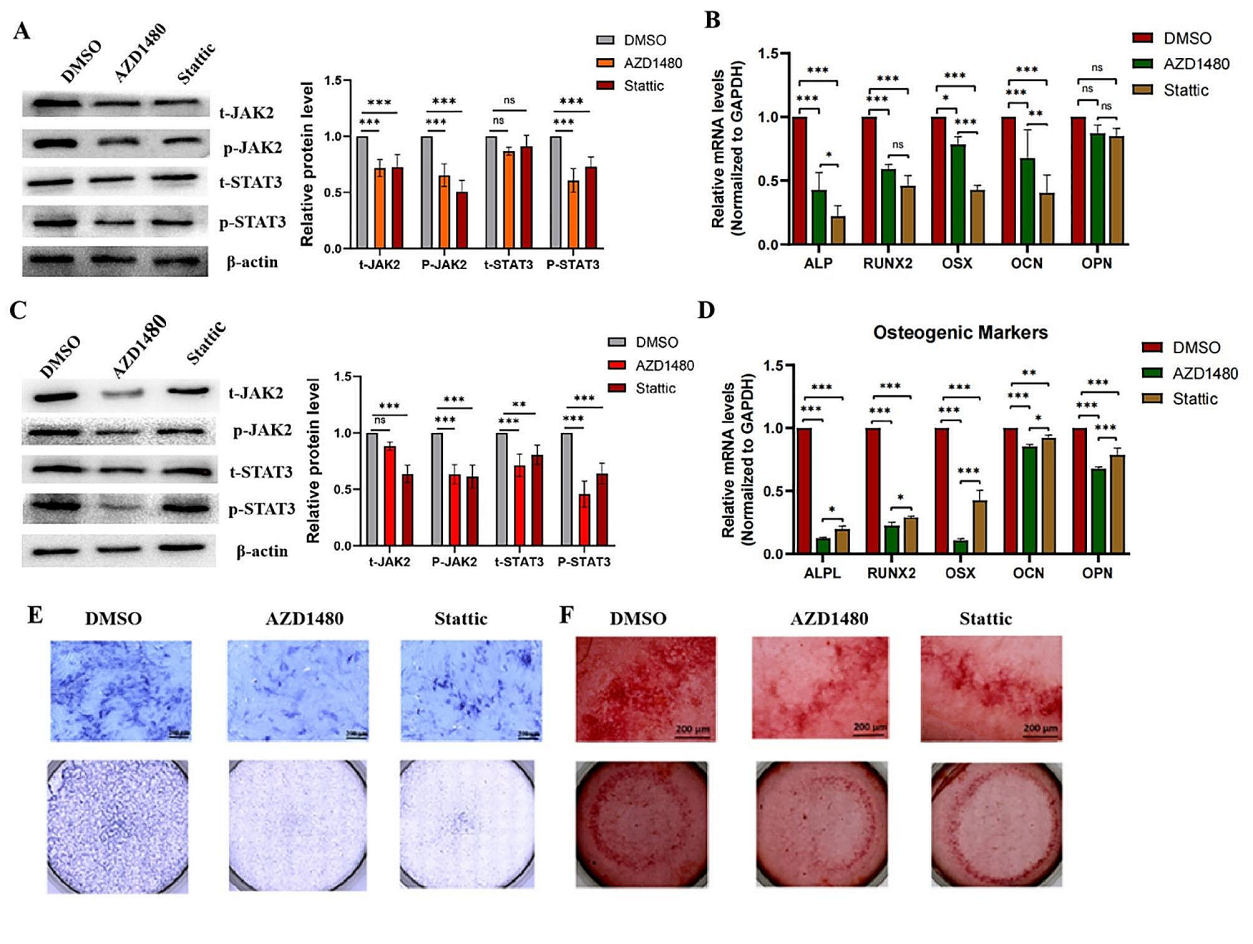
OSM promotes early and mid-stage osteogenic differentiation of tendon stem cells through the JAK2/STAT3 signalling pathway. (A, B,E)After 7 days of OSM-induced osteogenic differentiation in the presence of AZD1480 and stattic, alkaline phosphatase staining was performed. Scale bar represents 200 μm. Expression of p-JAK2 and p-STAT3 proteins as well as osteogenic marker genes was analysed by western blotting and qPCR(*n* = 3). One-way ANOVA. (C, D,F) After 14 days of OSM-induced osteogenic differentiation in the presence of AZD1480 and stattic, alizarin red staining was performed. Scale bar represents 200 μm. Expression of p-JAK2 and p-STAT3 proteins and osteogenic marker genes were analysed by western blotting and qPCR, respectively (*n* = 3). One-way ANOVA. Data are presented as means ± standard deviation. *p < 0.05; **p < 0.01; ***p < 0.001

**Fig. 5:**
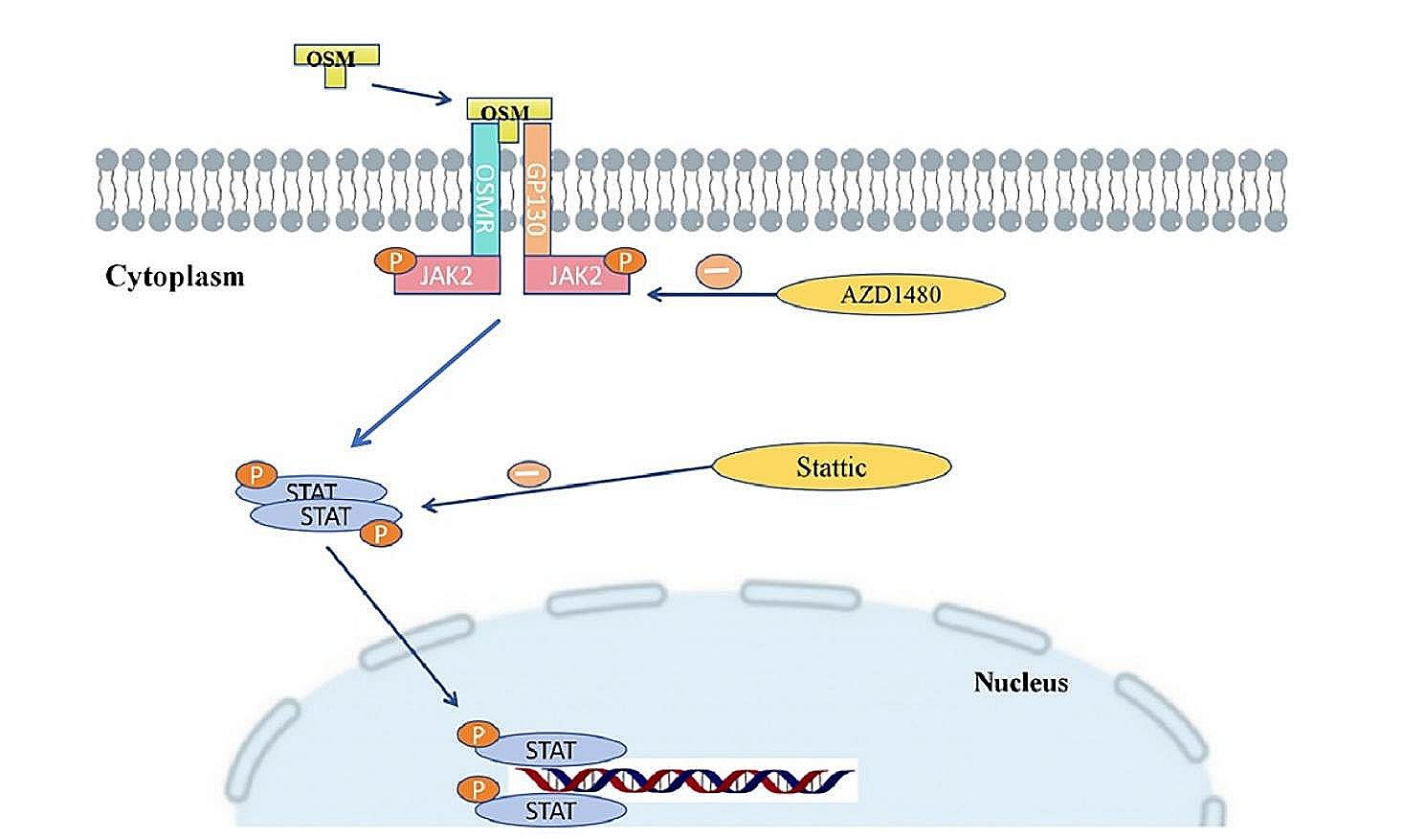
OSM promotes osteogenic differentiation of tendon stem cells through the JAK2/STAT3 signalling pathway. OSM binds to the tendon stem cell receptor OSMR/GP130, activating and phosphorylating JAK2. Phosphorylated JAK2 activates STAT3 downstream, and phosphorylated STAT3 forms a dimer that translocates to the nucleus and binds to DNA to regulate gene transcription. AZD1480 and stattic inhibit activation and phosphorylation of JAK2 and STAT3, respectively

To further explore the effect of the JAK2/STAT3 signalling pathway on osteogenic differentiation of TDSCs, we added JAK2/STAT3 signalling pathway inhibitors to the osteogenic differentiation medium. After osteogenic differentiation of TDSCs for 7 and 14 days, the expression of p-JAK2, p-STAT3, and osteogenic marker genes was detected by western blotting and qPCR. Compared with the DMSO control group, p-JAK2, p-STAT3, and osteogenic marker gene expression was reduced in both inhibitor groups (AZD1480 and stattic) (Fig. 4A, B and C, and 4D). Additionally, after 7 days of osteogenic differentiation, alkaline phosphatase staining in AZD1480 and stattic groups was lower than that in the DMSO control group (Fig. 4E). Alizarin red staining of TDSCs after 14 days of osteogenic differentiation indicated reduced mineralisation in AZD1480 and static groups compared with that in the DMSO control group (Fig. 5F).

These results showed that OSM promoted early and mid-stage osteogenic differentiation of TDSCs through the JAK2/STAT3 signalling pathway.

## Discussion

After tendon injury, stem cells within the tendon tissue can differentiate normally into tendon cells for repair [15, 16]. However, the repair capacity is limited and most injuries develop tendon heterotopic ossification.Tendon heterotopic ossification is a chronic inflammatory disease in which TDSCs are induced to differentiate toward osteoblasts in the chronic inflammatory environment. In this study, we demonstrated that OSM promoted osteogenic differentiation of TDSCs through the JAK2/STAT3 signalling pathway in vitro.

TDSCs act as osteogenic precursor cells and play a major role in heterotopic ossification [17, 18]. Additionally, OSM promotes osteogenic differentiation of cells. Guhard et al. [19]. found that OSM promotes osteogenic differentiation of MSCs and that neutralisation of OSM or OSM receptor subunits and OSMR attenuates osteogenic differentiation. M1-type macrophages secrete OSM through the COX-2/PGE_2_ pathway, which in turn promotes osteogenic differentiation of MSCs, and the osteogenic differentiation effect is weakened by COX-2 inhibitors such as celecoxib and meloxicam [20]. Our study showed that OSM promoted osteogenic differentiation of TDSCs, which is consistent with the results of previous studies. ALP was mainly expressed in the early stage of osteogenic differentiation, and during osteogenic differentiation, ALP was gradually downregulated, and expression of OCN and OPN was increased [21, 22]. During osteogenic differentiation of TDSCs, 7 and 14 days represented early and mid-stages, respectively, and production of ALP in the early stage was higher than that of OCN and OPN, and production of ALP in the mid-stage was lower than that of OCN and OPN.

JAK/STAT is an intracellular signal transduction pathway that participates in various biological processes such as cell proliferation, differentiation, haematopoiesis, apoptosis, and immunoregulation [23–25]. The JAK/STAT signalling pathway also has an important role in osteogenic differentiation. Yang et al. [26]. found that OSM secreted by macrophages binds to the GP130/OSMR receptor complex, which activates JAK1/2 and mediates phosphorylation of STAT3 to promote osteogenic differentiation of MSCs, and that JAK1/2 inhibitors weaken osteogenic differentiation. Additionally, Yang et al. [13]. found that JAK2/STAT3 inhibitors significantly reduce the incidence of heterotopic ossification during OSM-induced differentiation of ligamentum flavum cells to osteoblasts in vitro. Zhang et al. [27]. also noted that blockade of the JAK signalling pathway diminishes leptin-induced osteogenic differentiation. Our results showed that JAK2 and STAT3 inhibitors attenuated osteogenic differentiation of tendon stem cells, and p-JAK2 and p-STAT3 expression levels as well as alkaline phosphatase and alizarin red staining were reduced by various degrees, a result that was consistent with those of the previous study.

OSM promotes osteogenic differentiation of tendon stem cells, mainly through binding to OSMR/GP130 receptors on the cell membrane, intracellular JAK2/STAT3 is activated and phosphorylated successively, and the phosphorylated STAT3 forms a dimer that transfers to the nucleus and binds to the corresponding DNA, which in turn leads to osteogenic differentiation. Among them, AZD1480 and Stattic can block the activation and phosphorylation of JAK2 and STAT3 respectively **(**Fig. 5**)**. The present study also has shortcomings. OSM promotes osteogenic differentiation of tendon stem cells through multiple signalling pathways, only the JAK2/STAT3 signalling pathway was investigated in this experiment, and other signalling pathways need to be explored in further studies.

## Conclusions

OSM promotes osteogenic differentiation of TDSCs through the JAK2/STAT3 signalling pathway. This finding improves our understanding of the pathogenesis of tendon heterotopic ossification and provides new ideas for treatment of this disease.The progression of tendon heterotopic ossification or even reversal of the disease can be prevented by administration of OSM antibodies or JAK2/STAT3 signalling pathway inhibitors.

## Data Availability

No datasets were generated or analysed during the current study.
